# Changes in Sexual Fantasy and Solitary Sexual Practice During Social Lockdown Among Young Adults in the UK

**DOI:** 10.1016/j.esxm.2021.100342

**Published:** 2021-05-05

**Authors:** Cory J. Cascalheira, Mark McCormack, Emma Portch, Liam Wignall

**Affiliations:** New Mexico State University, Las Cruces, NM, USA; University of Roehampton, London, England, UK; Bournemouth University, Poole, England, UK; Bournemouth University, Poole, England, UK

**Keywords:** COVID-19, Desire, Fantasy, Lockdown, Masturbation, Pornography, Sexual practice

## Abstract

**Introduction:**

Pandemic-related social lockdown limited many sexual behaviors, but to date, no study has examined the perceived impact of social lockdown due to COVID-19 on sexual fantasy and solitary sexual behavior.

**Aims:**

The present study sought to examine the perceived impact of social lockdown on sexual fantasy and solitary sexual behavior among UK young adults in various living situations.

**Methods:**

A convenience sample of 565 adults aged 18–32 and living in the UK completed anonymous, web-based, study-specific questionnaires between May 14 and 18, 2020, 7 weeks after social lockdown was initiated. Mixed-method analyses were conducted.

**Main Outcome Measures:**

The study presents qualitative and quantitative data. Criterion variables were measured dichotomously as increases (vs no change) in sexual fantasy and increases (vs no change) in pornography consumption. Predictor variables were living arrangement, relationship status, and postlockdown changes in masturbation and pornography consumption.

**Results:**

Of all, 34.3% engaged in more sexual fantasizing during lockdown; women were more likely than men to report this increase. Living context and relationship status were predictors of increased fantasizing. Of all, 30.44% reported an increase in at least one solitary sexual practice. This increase was associated with an increase in sexual fantasizing and also with increased pornography consumption. Nineteen percent of participants reported an increase in pornography use, with men being more likely than women to report this increase. Participants mostly attributed their increases to boredom, increased free time, and replacing partnered sex.

**Conclusion:**

Shifts in sexual fantasizing and solitary sexual practices were predicted by living arrangements, relationship status, and gender. The present findings suggest that the assessment of sexual fantasy and solitary sexual activities may benefit patients presenting with pandemic-related stress. Although mostly exploratory, significant changes in sexual fantasy and solitary sexual practices were observed. A cross-sectional design, convenience sampling, and study-specific measures are limitations.

## Introduction

The COVID-19 pandemic resulted in physical distancing measures in the United Kingdom (UK), known as social “lockdown,” between March 16, 2020, and May 13, 2020, which was repeated in November 2020 and January 2021. During lockdown, physical interactions were limited to members of one's household,^1^ implicitly banning sexual interactions between people who did not live together. During the first lockdown, many young adults (eg, university students) returned to live with parents in large numbers due to the Coronavirus Act 2020.^2^^,^^3^ These social changes have the potential to impact people's sex lives and wellbeing,^4–7^ possibly affecting partnered people and women differently from single people and men,^8–10^ especially if living arrangements changed.^11^

Limited research has examined the impact of social lockdown on sexuality.^9^^,^^12–15^ Studies have focused on changes in sexual behaviors, with evidence of a decline in frequency of sexual behaviors in the United States and China^12^^,^^13^ and an increase in frequency of sexual activities in the UK.^9^ Sexual repertoires expanded for a minority of participants, including a rise in pornography consumption.^12^ There is also evidence that relationship context can have diverse influences in the time of COVID-19, such as higher levels of emotional wellbeing among married couples.^10^ Since younger adults are less likely to be in co-habiting romantic relationships and may have changed their living arrangements during social lockdown,^3^^,^^16^ and given the importance of these variables in partnered sex,^9^^,^^12^^,^^13^ relationship status and living arrangement may be important predictors of sexual fantasy and solitary sexual behavior during lockdown.

However, changes in sexual fantasy and solitary sexual practices during lockdown have not been investigated. These are important aspects of sexuality, particularly when facing restrictions on personal freedoms, yet both masturbation and pornography can be stigmatized.^17–19^ Sexual fantasy and solitary sexual practices can be used as mechanisms to cope with stressful situations,^18^ yet research has traditionally focused on high-risk sexual activities and illegal behaviors in this context.^19^^,^^20^ However, a recent move to considering sexual activity and pornography use as leisure activities in the general population is gaining credence.^21–23^ From this sex-positive perspective,^4^^,^^24^^,^^25^ understanding the impact of social lockdown on young adults’ sexual fantasies and solitary sexual behaviors is an important issue.

Therefore, given the rapid emergence of COVID-19 and the sudden changes to social routines, the present study used a series of ad hoc questionnaires to describe categorical changes in sexual fantasies and solitary sexual practices during the peak of social lockdown in the UK. A mixed-method approach was used. Three hypotheses (H) were specified and tested:

**H1:** Increases in sexual fantasizing will be associated with (a) relationship status and (b) living arrangement.
**H2:** Increases in sexual fantasizing will be associated with increases in: (a) solitary masturbation; and (b) solitary pornography use;
**H3:** Increases in solitary pornography use will be associated with increased solitary masturbation.

## Materials And Methods

### Participants

Inclusion criteria were (1) live in the UK and (2) be an adult between the ages of 18 and 32. This age range was selected because there is evidence that young adults report more stress in relation to COVID-19, and as a group they have received less scholarly attention despite facing many transitions and uncertainties related to sex and sexuality.^26^^,^^27^ Data were from 565 participants drawn from an anonymous, cross-sectional survey conducted to explore sexual health and wellbeing during social lockdown.^28^ On average, participants were in their twenties ($M_{\text{age}}$  = 25.41, *SD* = 4.15) who did not tend to self-isolate (69% no, 31% yes) and were not working as essential workers (78.6% no, 21.4% yes) at the time of data collection. Participants rated their perceived global health status on a scale of 1 (*poor*) to 5 (*excellent*), reporting above-average health (*M* = 3.52, *SD* = 0.91). See Table 1 for additional demographic features of the dataset.

**Table 1:** Sociodemographic characteristics of participants (N = 565)

Variable	*n*	%
Gender		
Cisgender woman	338	59.8
Cisgender man	220	38.9
Non-binary	5	0.88
Unknown	2	0.35
Sexual Orientation		
Heterosexual	446	78.9
Bisexual	52	9.2
Mostly heterosexual	41	7.26
Homosexual	20	3.54
Mostly homosexual	6	1.06
Ethnicity		
White British	409	72.4
White other	55	9.7
South Asian	38	6.7
Mixed ethnicity	28	5.0
African/Caribbean	18	3.2
Other Asian	15	2.7
Other ethnicity	2	0.35
Relationship Status		
Serious	341	60.4
Single	184	32.6
Casual	38	6.7
Unknown	2	0.35
Currently Living With		
Partner	234	41.4
Family or children	214	37.9
Friends or others	58	10.3
Alone	37	6.5
Other	22	3.9

*Note*: Percentages may not total 100% due to rounding. Self-reported ethnicity and current living situation were condensed from additional categorical choices in the original survey.

### Procedure

Participants were recruited from *Prolific,* an online participant recruitment site. *Prolific* is an alternative to Amazon Mechanical Turk and yields high-quality data; it also prevents multiple completions by the same person.^29^ After providing informed consent digitally, participants completed a demographic questionnaire and descriptive survey items in Qualtrics. Data collection occurred between May 14 and 18, 2020, 7 weeks after social lockdown was initiated in the UK. No identifying information was collected. Survey data were stored on password-protected computers and encrypted servers. Ethical approval was granted from Bournemouth University. Participants were reimbursed £3.75 for completion of the survey, an amount used to facilitate survey completion that is not considered coercive.^30^ Identifying information used for compensation was managed by *Prolific*, thereby protecting participant confidentiality.

### Measures

Three ad hoc questionnaires, a method which has been used successfully in other rapid-deployment research on COVID-19,^31^^,^^32^ were used in the present analyses.

#### Solitary Sexual Behaviors

Participants were asked to indicate whether they engaged in solitary masturbation, solitary pornography viewing, and solitary sex toy use prior to lockdown and during lockdown. Responses were measured dichotomously. Affirmative responses prompted participants to indicate whether they had engaged in the sexual behavior more or less during lockdown.

#### Sexual Fantasies

Participants reported whether they had experienced increased levels of sexual fantasizing during lockdown (eg, “Have you started sexually fantasizing more since social lockdown?”) using a dichotomous response scale. If so, they were prompted to explain how the nature of their fantasies had changed (eg, “Please describe how your fantasies have changed”).

#### Pornography Consumption

Participants were asked a series of retrospective questions specific to their pornography use. If participants indicated a change in pornography use (eg, “Has the amount of pornography viewed during social lockdown changed?”), then they were prompted to explain their answer in a free text box. Participants also selected the ways in which their pornography had changed from a list of study-specific behaviors (eg, “I watch porn when bored,” “I watch less porn,” etc.). Finally, they indicated whether their partners knew about their pornography use and, if not, were prompted to explain why in a free text box.

### Data Analysis

All analyses were conducted using R Studio 1.3.1056.^33–35^ For quantitative analyses, Pearson chi-square contingency table analyses were conducted to examine both hypotheses. Chi-square contingency table analyses were selected because (1) hypotheses implied tests of association, (2) data were at the nominal level of measurement, and (3) at least one variable in each analysis was not dichotomous. Missing values were removed from all chi-square analyses. After each contingency table analysis, post-hoc pairwise comparisons using Bonferroni corrections were conducted to evaluate differences among the proportions.^36^^,^^37^ This procedure enabled the interpretation of chi-square results. Additionally, post-hoc gender analyses using Fisher's exact, 2-tailed probability test (without Yates continuity correction) were conducted. Power analyses, conducted in G*Power 3.1.9.4,^38^ are reported below. For qualitative analyses, a modified version of conventional content analysis^39^ and functions from the R package *tidyverse*^40^ were used. The general structure of the R command^35^ involved a conditional statement, string detection, and regular expressions to generate categories,^41^ a level of content analysis that sufficiently captured overall meaning.^39^

## Results

### Increases In Sexual Fantasy During Lockdown

Of all, 34.3% of participants reported an increase of sexual fantasizing during lockdown. Of the 194 participants who reported an increase in sexual fantasizing, 43.8% were cisgender men and 55.2% were cisgender women. The gender difference was significant, $\chi^{2}$(1) = 4.99, *P*= .025. Among those reporting an increase, 186 participants (95.9%) provided a reason for the change (see Table 2 ).

**Table 2:** Self-reported reasons for how sexual fantasizing changed during social isolation (n = 186)

Code	%	Example
Changes in Sexual Fantasizing		
More Frequent	43.5	“They have become more frequent, and more of a regular escape.”
More Intensity or Variety	24.7	“I fantasize more and more vividly, result of reading more sexual stories, more rough and kinky than usual.”
About Non-Partner	17.7	“They haven't changed so much—but I get pangs for different people in my past life. It's like my memories are more vivid again as my brain isn't rushing about all day. So, I'll have fantasies again for a teacher I had at school 15 years ago. But it lasts a few days then goes to the next.”
About Primary Partner	12.4	“I am fantasising more during lockdown, I believe this is due to less sexual interaction with my partner and the detrimental effect isolation has on myself.”
No Change in Fantasy Type	5.91	“They haven't changed […]”
Sex in Public	2.15	“I'm imagining doing it outside.”
Reasons for Changes		
Coping with Free Time	17.2	“I am more easily distracted, because I am bored, and so I fantasise about actors in series I am watching (for example)”
Desiring Intimacy	5.38	“I have been fantasising in a more romantic way than before, I miss my partner.”
Other Reason	4.30	“Just want a shag.”
Unsure	1.08	“I don't know.”

*Note*: Percentages do not add up to 100% because an open-ended response could have been classified to more than one code.

The first 5 × 2 chi-square test of independence exploring increases in sexual fantasies and living arrangement was significant, $\chi^{2}$(4, 565) = 30.15, *P*< .001, Cramér's *V* = 0.23, and reached sufficient power (0.98). Thus, having increased sexual fantasies was associated with living arrangements. Participants living with children or family members were more likely to report no changes (53.74%) rather than increases (46.26%) in sexual fantasizing, *P*< .001; participants living with partners were also more likely to report no changes (77.35%), versus increases (22.65%), in sexual fantasizing.

As shown in Table 3 , 4 of the 10 standardized residuals significantly contributed to the omnibus chi-square statistic. Young adults living with children or family members reported no change in sexual fantasizing significantly less than expected by chance, but reported an increase in sexual fantasizing significantly more than expected. Additionally, young adults who were living with sexual partners reported no change in sexual fantasizing significantly more than expected. They reported an increase in sexual fantasizing significantly less than expected.

**Table 3:** Chi-square, standardized residuals, and relative and absolute contributions of sexual fantasizing by living arrangement

Cell	$f_{\text{rc}}$	Cell Chi-square	Standardized residual	Relative contribution %
Living with children or family				
Increase	99	8.758	4.638*	29.045
No change	115	4.592	− 4.638*	15.229
Living with friends or others				
Increase	22	0.211	0.5981	0.698
No change	36	0.110	− 0.5981	0.366
Living alone				
Increase	15	0.553	0.949	1.834
No change	21	0.29	− 0.949	0.962
Living with partner				
Increase	53	9.388	− 4.945*	31.136
No change	181	4.923	4.945*	16.326
Some other arrangement				
Increase	5	0.871	− 1.175	2.889
N o change	17	0.457	1.175	1.515

⁎ *P* < .001.

The second 3 × 2 chi-square test of independence exploring increases in sexual fantasizing and relationship status was marginally significant, $\chi^{2}$(2, 562) = 6.45, *P*= .04, Cramér's *V* = 0.11, and slightly underpowered (.64). Thus, results suggested that sexual fantasizing was associated with one's relationship status, albeit weakly.^37^ None of the post-hoc comparisons using standardized residuals were significant. Thus, these results partially support H1.

### Increases In Solitary Sexual Activity During Lockdown

During social lockdown, increases in solitary masturbation (25.66%), watching porn alone (19.47%), and using a sex toy alone (8.5%) were reported. Of all, 30.44% of participants reported an increase in at least one solitary sexual behavior, 14.69% reported an increase in two solitary sexual behaviors, and 4.25% reported an increase in all three solitary sexual behaviors. Among those who reported an increase in at least one solitary sexual behavior, 52.9% were cisgender men and 45.3% were cisgender women. The gender difference was not significant, $\chi^{2}$(1) = 1.97, *P*= .161.

The third 2 × 2 chi-square test of independence exploring sexual fantasies and solitary masturbation was significant, $\chi^{2}$(1, 492) = 64.417, *P*< .001, Cramér's *V* = 0.36, and had sufficient power (1.0). Young adults who reported more sexual fantasies also increased their amount of solitary masturbation during social lockdown significantly more than expected, *P*< .001. Therefore, hypothesis H2a was supported.

The next 2 × 2 test of independence exploring sexual fantasizing and solitary pornography use was significant, $\chi^{2}$(1, 395) =38.82, *P*< .001, Cramér's *V* = 0.31, and sufficiently powerful (0.99). Young adults who sexually fantasized more often during social lockdown also watched more pornography significantly more than expected, *P*< .001. For young adults who reported no change in sexual fantasizing, their increases in pornography use were significantly less than expected by chance alone, *P* < .001. Thus, there was evidence to support hypothesis H2b.

Finally, a 2 × 2 test of independence exploring solitary pornography use and solitary masturbation was significant, $\chi^{2}$(1, 389) = 189.16, *P*< .001, Cramér's *V* = 0.70, and achieved adequate power (1.0). Young adults who reported watching more pornography alone also increased their solitary masturbation significantly more than expected, *P*< .001. Among young adults who reported some other change in their pornography consumption during social lockdown (see Table 4 ), their reported increases in masturbation were significantly less than expected, *P*< .001. Therefore, hypothesis H3 was supported.

**Table 4:** Changes in patterns of pornography use during national lockdown (N = 565)

Variable	*n*	%
Solitary Use	**317**	**56.1**
While masturbating	126	22.3
Watched alone	100	17.7
Only when bored	91	16.1
Consistent Use	**241**	**42.7**
Never watched	229	40.5
Remained the same	12	2.1
Directional Changes	**174**	**30.8**
Watched less	87	15.4
Quicker to watch*	33	5.8
Watched for longer	33	5.8
Watched less variety	20	3.5
Rarely watches	1	.18
New Behaviors	**75**	**13.3**
Watched different types	51	9.0
Watched with partner	20	3.5
Stopped watching	2	.35
Watched during sex	1	.18
Watched live cams	1	.18

*Note*: Percentages do not equal 100% since participants could select more than one behavior. Boldface represents aggregate counts and frequencies for each superordinate category.

⁎ Represents the affirmative responses to the statement, “I am quicker with my porn use” which, in a UK context, suggested using pornography more readily than before social lockdown.

### Exploring Changes In Pornography Consumption

Frequencies of specific pornography viewing habits are shown in Table 4. Proportional data indicated that 59.5% of the sample watched pornography during social lockdown. Among participants with partners, 66.7% reported that their partner knew about their pornography use. In terms of changes in pornography consumption, 64.1% reported no change, 19% reported an increase, and 17.2% reported a decrease. Among participants reporting an increase, 64.8% were cisgender men and 33.3% cisgender women. The difference in proportion between men and women was significant, $\chi^{2}$(1) = 20.75, *P*< .001. Among participants reporting a decrease, 57.7% were cisgender women, 42.3% were cisgender men, and the difference was significant, $\chi^{2}$(1) = 4.64, *P*= .031. Participants reported reasons for the changes in pornography consumptions (see Table 5 ).

**Table 5:** Rationales for changing pornography consumption

Reasons for increase (*n* = 102)	%	Reasons for decrease (*n* = 96)	%
1. Cope with boredom	37.3	1. Lack of alone time	53.1
2. Cope with free time	23.5	2. Lower sex drive	15.6
3. Replace partnered sex	13.7	3. Described frequency	13.5
4. Relieve stress	7.84	4. Stated porn was “addictive”	6.25
5. Watch with partner	4.9	5. Unsure	6.25
		6. Busier than usual, no time	5.21
6. Other (eg, “more horny now”)	4.9	7. Lost interest in porn	4.17
7. Described frequency	2.75	8. Other (e.g., “websites blocked”)	3.1

## Discussion

In this study, we examined the impact of social lockdown on young adults’ sexual fantasies and solitary sexual acts. Drawing on a survey of 565 British adults aged 18–32 collected at the peak of social lockdown restrictions, we found that social context had a significant impact on changes in sexual fantasy; people living with children or other family members were more likely to see an increase in sexual fantasizing and relationship status predicted this change, but weakly. Just under one-third of participants reported an increase in at least one solitary sexual practice with one quarter increasing in solitary masturbation. Increased sexual fantasies were associated with increases in solitary masturbation as well as solitary pornography consumption. While pornography was consumed for several reasons, a strong effect was observed in the association between solitary pornography and solitary masturbation specifically. Concealment of pornography use from a partner and increased solitary sexual activities approached an even gender split, although there was insufficient power to apply a gender analysis to the chi-square statistics. Finally, the frequencies of pornography consumption and the increases in sexual fantasizing by gender were significant: men reported greater increases in pornography use than women; women reported greater increases in sexual fantasizing than men.

Several research and clinical implications are evident. In respect to research implications, the current findings call for greater attention to sexual fantasy and solitary sexual activity during periods of social change. These findings regarding limited but significant changes in sexual practice are similar to other work.^12^ However, our focus on solitary sexual practice highlights a rise in masturbation and the consumption of pornography, but in moderate ways. Importantly, the language of pornography addiction was only used by respondents who reported a *decrease* in pornography consumption during lockdown. Thus, contrary to dominant narratives of the harms of pornography consumption,^17^ solitary masturbation and pornography consumption could be more appropriately considered a leisure activity during a period of heightened restrictions on personal activities.^22^ Framing increases in solitary sexual behavior as leisure fits with the broader sex-positive approach that is gaining traction in sexology and sexual medicine,^25^^,^^42^ which can be useful in patient-centered care and rapport-building.^25^ Nonetheless, this implication is speculative because our findings are exploratory and need further study. Regarding gender differences, the greater increase in pornography use among men compared to women is consistent with other findings^9^; however, the greater increase is sexual fantasizing among women, compared to men, could suggest that women experience sex during the social lockdown in a more nuanced way than previously reported.^9^^,^^13^

In terms of clinical implications, the assessment of sexual fantasy and solitary sexual activities may benefit patients presenting with pandemic-related stress. Patients may feel guilty or ashamed about their sexual fantasies while spending greater time with their families, but the present findings suggest that the association may be normative, so psychoeducation in this area could alleviate emotional distress. Similarly, for men who perceive their pornography use to be problematic,^43^ clinicians might reduce sexual shame, engage patients in motivational interviewing, and facilitate insight into use patterns by discussing the reasons for increased pornography consumption presented in this study. For women with low sexual desire during COVID-19,^13^ the positive association between sexual fantasy and solitary masturbation could be used to help these patients achieve arousal, such as suggesting fantasies that was pleasurable before the pandemic.^44^

### Limitations

Results should be considered in light of several limitations. First, this study employs a cross-sectional design, recruiting participants during the peak social lockdown restrictions. As such, follow-up research is needed to explore the consequence of these changes in sexual practices due to social lockdown, especially when restrictions are eased or reinstated. Second, the study uses ad hoc questionnaires which, while useful in sudden social upheaval,^31^ are not standardized. Therefore, future studies on solitary sexual practices should use validated, reliable measures, such as the Sexual Fantasy Questionnaire,^45^ to extend the present findings. Third, since the study-specific questions used retrospective questions, response bias may have occurred, and the results should be considered exploratory. Fourth, the sample is limited to predominantly white, heterosexual young adults, recruited through a convenience sample that is not representative of the UK population. Further research needs to explore the experiences of sexual and ethnic minorities, as well as other age groups. Finally, while our gender analysis of descriptive statistics suggested little gender difference, more sophisticated analyses of gender as a variable is necessary where there are sufficient power and tenable parametric assumptions to do so.

## Conclusion

This study found that social lockdown had a significant impact on young adults’ sexual fantasizing and solitary sexual practices, and these changes were predicted by living arrangements, relationship status, and gender. Descriptive results show commonly reported reasons for changes in sexual fantasy and pornography consumption.

## Statement Of Authorship

Conceptualization, L.W., E.P., and M.M.; Methodology, L.W., and E.P.; Formal Analysis, C.C.; Writing - Original Draft, M.M., C.C., and L.W.; Writing – Review & Editing, M.M., C.C., L.W., and E.P.; Funding Acquisition, L.W., and E.P.

## Notes

*Funding* This work was supported by Bournemouth University.
